# Arthroscopic Fixation of Cell Free Polymer-Based Cartilage Implants with a Bioinspired Polymer Surface on the Hip Joint: A Cadaveric Pilot Study

**DOI:** 10.1155/2014/717912

**Published:** 2014-08-28

**Authors:** Matthias Lahner, Christian Duif, Andreas Ficklscherer, Christian Kaps, Lukas Kalwa, Tobias Seidl

**Affiliations:** ^1^Department of Orthopaedic Sports Surgery, Ruhr-University Bochum, St. Josef-Hospital, Gudrunstrße 56, 44791 Bochum, Germany; ^2^Department of Orthopaedic Surgery, Physical Medicine and Rehabilitation, University Hospital of Munich (LMU), Campus Großhadern, Marchioninistraße 15, 81377 München, Germany; ^3^BioTissue Technologies GmbH, Charitéplatz 1, 10117 Berlin, Germany; ^4^Tissue Engineering Laboratory, Department of Rheumatology and Clinical Immunology, Charité-Universitätsmedizin Berlin, Charitéplatz 1, 10117 Berlin, Germany; ^5^Westphalian Institute for Biomimetics, Westphalian University of Applied Sciences, Münsterstraße 265, 46397 Bocholt, Germany

## Abstract

This study investigates the adhesion capacity of a polyglycolic acid- (PGA-) hyaluronan scaffold with a structural modification based on a planar polymer (PM) surface in a cadaver cartilage defect model. Two cadaver specimens were used to serially test multiple chondral matrices. In a cadaver hip model, cell free polymer-based cartilage implants with a planar bioinspired PM surface (PGA-PM-scaffolds) were implanted arthroscopically on 10 mm × 15 mm full-thickness femoral hip cartilage lesions. Unprocessed cartilage implants without a bioinspired PM surface were used as control group. The cartilage implants were fixed without and with the use of fibrin glue on femoral hip cartilage defects. After 50 movement cycles and removal of the distraction, a rearthroscopy was performed to assess the outline attachment and integrity of the scaffold. The fixation techniques without and with fibrin fixation showed marginal differences for outline attachment, area coverage, scaffold integrity, and endpoint fixation after 50 cycles. The PGA-PM-scaffolds with fibrin fixation achieved a higher score in terms of the attachment, integrity, and endpoint fixation than the PGA-scaffold on the cartilage defect. Relating to the outline attachment, area coverage, scaffold integrity, and endpoint fixation, the fixation with PGA-PM-scaffolds accomplished significantly better results compared to the PGA-scaffolds (*P* = 0.03752, *P* = 0.03078, *P* = 0.00512, *P* = 0.00512). PGA-PM-scaffolds demonstrate increased observed initial fixation strength in cadaver femoral head defects relative to PGA-scaffold, particularly when fibrin glue is used for fixation.

## 1. Introduction

In the regenerative cartilage surgery, many surgical techniques were developed for coating focal articular cartilage defects. Current available surgical cartilage treatments include lavage and debridement, subchondral penetration procedures (microfracture technique, drilling, or abrasion chondroplasty), autogenous osteochondral transplantation (AOT), and autologous chondrocyte implantation (ACI) with or without using a scaffold [1–7].

As a single-stage procedure, the most used treatment of focal cartilage defects represents the microfracture (Mfx) technique in which a penetration of the subchondral bone layer is performed with subsequent effluent of progenitor cells from the bone marrow into the articular cartilage lesion [1, 2, 8]. The progenitor cells are similar to chondrocytes but form a fibrocartilaginous tissue which is mechanically and biochemically substandard to the originary cartilage tissue [9].

Scaffold-assisted single-step techniques were developed to enhance cartilage tissue regeneration by the combination of the Mfx with a biomaterial. By combination of the Mfx with a scaffold, the mesenchymal stem cells (MSCs) are attracted into the biomaterial and the chondrogenic differentiation of the MSCs to cartilage repair tissue is better assured such as the sole application of the Mfx [10, 11]. However, the fibrin clot is not mechanically stable, so the implanted scaffold enhances the mechanical stability in the defect zone additionally [12]. The biomaterial serves as a biostructure for cell attachment of the MSCs [13]. Different matrices are currently available for surgical cartilage repair like scaffolds with porcine collagen I/III membrane [14, 15]. Another scaffolding for cartilage repair is a cell free matrix composed of an absorbable nonwoven polyglycolic acid (PGA) textile combined with hyaluronic acid (HA) [16, 17]. The mechanical stability of the matrix allows for easy treatment and safe fixation in the cartilage lesion by fibrin sealant, cartilage, or transosseous suture, or by resorbable pins [18, 19]. However, the arthroscopic pin insertion for fixation of the scaffold represents a challenge during arthroscopic cartilage repair. An incorrect placed pin can release cartilage damage of the articular opposite side [20].

The importance of the primary fixation of the scaffolds is common, but there is no study available which examines the influence of a structural modification of the scaffold surface in order to achieve a higher adhesion ability [21].

Here we compare and quantify the primary fixation stability of a commonly used scaffold for cartilage repair attached by a planar bioinspired polymer (PM) surface in a cadaver hip model. The aim of our experimental study was that the arthroscopic implantation of a cell free polymer-based scaffold with a structure derived from tree-frog footpads was more stabile compared to an unprocessed scaffold.

## 2. Materials and Methods

### 2.1. Testing Protocol

**Figure 1:**
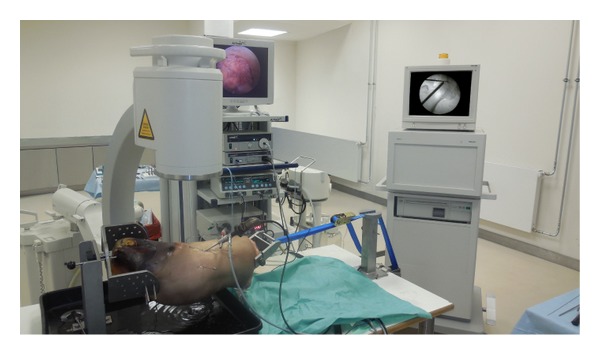
Experimental setup with arthroscopy and fluoroscopy equipment.

**Figure 2:**
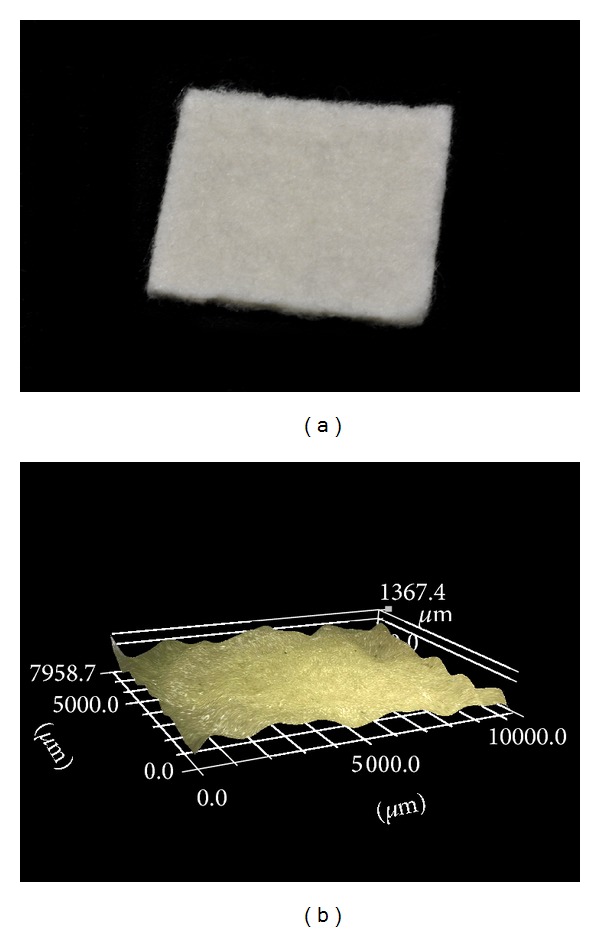
Polyglycolic acid- (PGA-) hyaluronan scaffold (a) and confocal stereomicroscopic picture of the scaffold before fixation (b).

**Figure 3:**
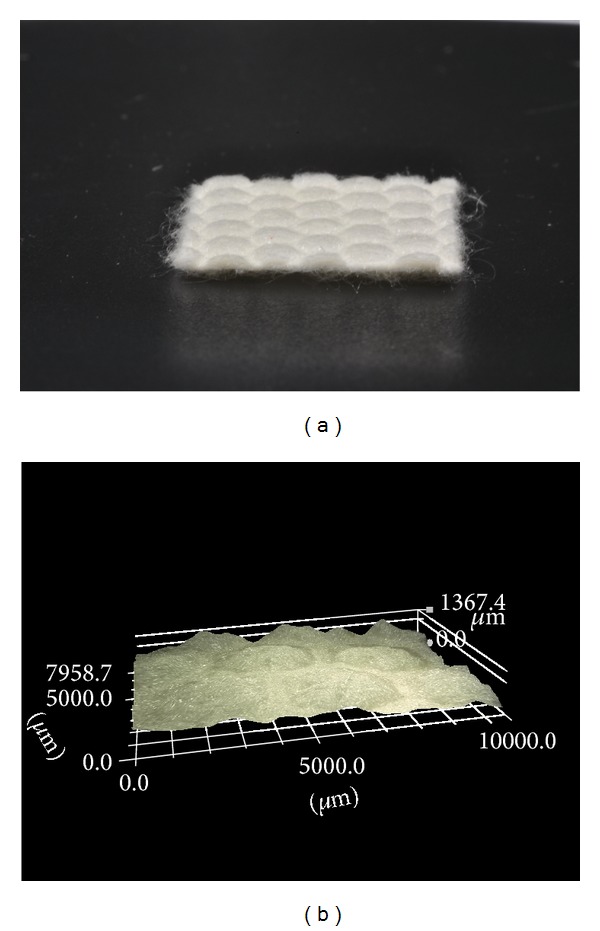
PGA-scaffold with polymer (PM) surface (a) and confocal stereomicroscopic picture of the scaffold before fixation (b).

The study was approved by the local ethical committee of the Ruhr-University Bochum (registration number 4611-13). The surgery was performed by two orthopaedic surgeons experienced in hip arthroscopy (Matthias Lahner, Christian Duif). The fluoroscope was placed diagonally across the table (Philips, Hamburg, Germany). In this study, two right human cadaver hips were arranged for hip arthroscopy in a supine position demonstrated in Figure 1. Arthroscopy for the central joint compartment was performed with distraction. The ventral portal was created under fluoroscope. The hip capsule was widened through a 1.5 mm guide wire with sequential telescoping 4 mm and 6 mm portal dilators (Arthrex, Naples, FL, USA). After advancing a 5.5 mm fenestrated metal cannula into the joint, an arthroscopic evaluation was performed with a 70° 4 mm scope (Arthrex). A second instrumentation portal approach was established with a 14-gauge needle under arthroscopic and fluoroscopic view. The hip capsule was penetrated with a 4 mm dilator and an 8 mm × 4 cm pass port button cannula (Arthrex) which was positioned to create a stable portal. By using a bone cutter (Arthrex), a 10 × 15 mm full-thickness cartilage defect was created in the zone 2 of the femoral head (hip 1 and 2) described by Ilizaliturri et al. [22]. Then different scaffolds were placed into the defect, either a PGA-hyaluronan scaffold without structural modification (Figure 2) or a bioinspired PGA-PM-scaffold (Figure 3). Both groups were implanted with or without fibrin glue (Evicel, Omrix Biopharmaceuticals NV, Diegem, Belgium). Both cadavers had all four treatments.

### 2.2. Testing Groups

The resorbable PGA-hyaluronan scaffold (BioTissue Technologies GmbH, Freiburg, Germany) of 10 mm × 15 mm × 1.1 mm was impregnated with an embossing stamp. The embossing stamp consisted of a thermoplastic polymer (PM) surface composed of acrylnitrile butadiene styrene (ABS). The PM was designed of a moulding form with computer-aided design (CAD) software from a 3D printer (Dimension BST, Stratasys, Eden Prairie, USA). The PGA-hyaluronan scaffold was loaded with the PM embossing stamp with a defined 4 kilo force for about 20 minutes in a laboratory-type drying cabinet at 80°C. The PGA-PM-scaffold was produced only for experimental use. For the fixation, the scaffold was placed directly onto the defect of the subchondral bone without additional material.

### 2.3. Arthroscopic Implantation

**Figure 4:**
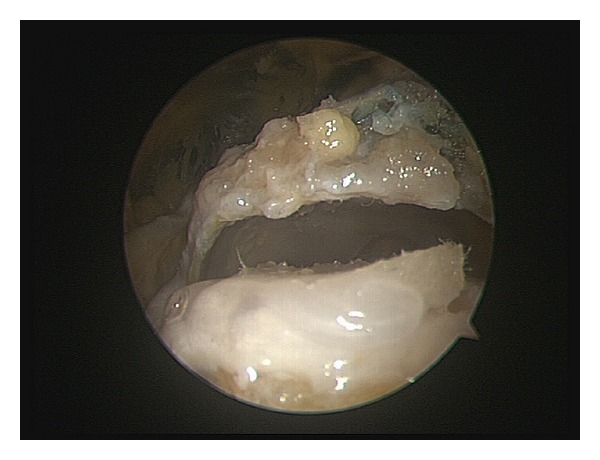
Implantation of the PGA-scaffold on a full-thickness femoral hip cartilage defect. The implant was used to cover the defect and fixed by a fibrin glue.

**Figure 5:**
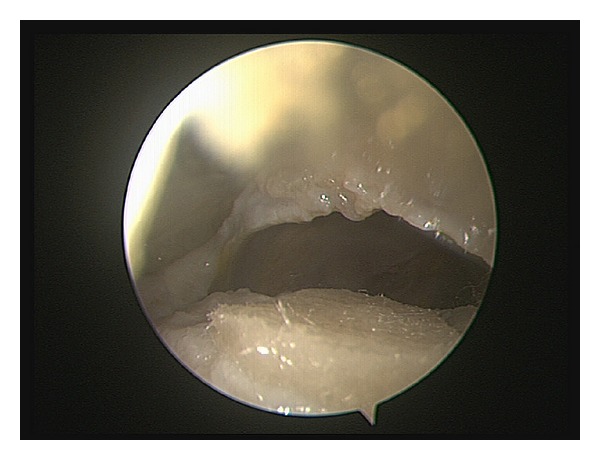
Implantation of the PGA-scaffold modified with a planar PM surface. The implant was used to cover the defect and fixed by a fibrin glue.

After evacuating the saline solution, the scaffold was implanted by the use of a tissue grasper and simply released above the full-thickness cartilage defect. By using a tissue elevator, the scaffolds were shaped into the defect to reach a 100% filling of the defect. Before fixation of the scaffold with fibrin glue, we used an application device with a 35 cm tip (Evicel) through the instrumentation portal. After removing the distraction, 50 cycles of flexion (90°) and extension (10°) were performed by moving the leg manually. The cycles were conducted by an independent third-party-assistant that was blinded to the assessment of the measurements. Then, the hip joint was refilled with saline solution and the defect area with the implanted scaffold was inspected (Figures 4 and 5). After the implantation of scaffold, the matrix was completely removed and the fibrin glue was eliminated by the shaver. All hip cadavers underwent the presented motion protocol with two fixation techniques tested on each hip. The outline attachment and the percentage of defect-covering scaffold were noticed as well as the integrity of the scaffold itself. The endpoint fixation strength was manually tested by a palpating hook. The properties of the scaffold were directly evaluated by two orthopedic surgeons. All scaffolds were photographed at each rearthroscopy and reviewed by the third collaborator who did not participate in the initial surgery. We performed therefore three measurements per fixation. A consensus of all three evaluators was taken as the end result.

### 2.4. Classification of Scaffold Evaluation

To classify the differences concerning adhesion and integrity between the tested scaffolds, we modified the scoring system developed by Bekkers et al. [23]. Outline attachment, area coverage, scaffold integrity, and endpoint fixation were assessed and a corresponding score was determined in a 5-point scale (Table 1).

### 2.5. Statistical Analysis

For each individual fixation technique, the average scores and standard deviations per scoring item were calculated after 50 motion cycles. The arithmetic mean value and the SD were calculated for the variables above and measured with Microsoft Excel (Microsoft, Redmond, WA, USA). The values were recorded in IBM SPSS Statistics 22 (PASW 22, SPSS Inc., Chicago, IL, USA). Mean values of each set were performed using Mann-Whitney* U* test with level of significance defined at *P < 0.05.

## 3. Results

All the fixation techniques were feasible through the initial incision. None of the specimens showed macroscopic damage at the opposing articular cartilage surface. We compared the unfixed PGA-scaffold versus the unfixed PGA-PM-scaffold plus the PGA-scaffold with fibrin glue versus the PGA-PM-scaffold with fibrin glue. The results of the scaffold fixation are shown in Table 2. Concerning the scaffolds fixed with fibrin glue, the fixation with PGA-PM-scaffolds accomplished significantly better results compared to the PGA-scaffolds relating to the outline attachment, area coverage, scaffold integrity, and endpoint fixation (*P* = 0.03752, *P* = 0.03078, *P* = 0.00512, *P* = 0.00512). Concerning the outline attachment and endpoint fixation, lower statistically significant scores were found for the unfixed PGA-PM-scaffold compared to the PGA-scaffold (*P* = 0.02034, *P* = 0.01314). With regard to the area coverage and scaffold integrity, no statistical difference was observed between the unfixed PGA-scaffold and the unfixed PGA-PM-scaffold after 50 cycles (*P* = 0.17384   *P* = 0.06576). Closer examination showed an incomplete detachment of the PGA-scaffold without fibrin glue to a complete attachment of the PGA-PM-scaffold with adhesive fixation. However, no rupture of the scaffold fiber was observed during arthroscopic implantation. Relating to the scaffold integrity, the PGA-PM-scaffold with fibrin glue achieved the best result after the motion cycles. The fibrin glue fixation with PGA-PM-scaffolds provided the best scaffold integrity as compared to scaffolds without adhesive fixation after 50 cycles. The endpoint fixation strength of the scaffold was highest in the PGA-PM-scaffold fixed with fibrin glue.  Table 3 shows a summary concerning the advantages and disadvantages as well as the pros and cons of the clinical procedure of the PGA-PM-scaffolds.

## 4. Discussion

In the present study, we assessed the primary arthroscopic fixation stability of scaffold-based tissue-engineered implants of full-thickness cartilage defects in cadaver hips to simulate the adhesion properties. Regarding the fixation, we showed that the adherence on the cartilage defect was higher with the PGA-PM-scaffold than with the unmodified PGA-scaffold. These results were collected in experimental settings with high similarity to the intended application. They confirm the initial assessment from the development process.

This study was limited by the reduced number of implanted scaffolds and the lack of power analysis. Another limitation of our study was that we did not apply static uniaxial tensile tests on* in situ* cadaver legs; thus the transfer of the findings to a clinical setting is difficult. However, we did not accomplish intra-articular pressure measuring, but it can be assumed that the anatomical precondition of the hip joint with additional burden during motion generated a shearing force on the scaffold, which is similar to the* in vivo *conditions. This shear force will partially be conveyed directly by friction with the opposing cartilage and partially by the intra-articular remaining saline solution. Further biomechanical studies are needed to prove whether a slightly submerged implant could protect it from the friction* in vivo*, although in one rabbit model trial, it was demonstrated that submerged metal grafts in focal cartilage lesion deranged the integrity of the articulating cartilage surfaces [24].

Bekkers et al. demonstrated the need for a compromise between fixation stability and scaffold integrity [23]. Therefore, the primary adhesion of the implanted scaffold without further fixation devices may help to eliminate this discrepancy and improve results of fixation of the scaffold which are implanted arthroscopically.

The present study examines for the first time different arthroscopic fixation techniques of cartilage implants of the hip joint in an experimental setup. It demonstrates that bioinspired structural alterations of scaffolds may support arthroscopic application significantly. Since the role of hip arthroscopy and consequently the number of surgeons who apply this technique have significantly increased in the recent years our study presents an important impulse towards easier procedures and subsequently broader clinical application [25].

Furthermore, a stickier scaffold may allow cartilage repair as a simple one step procedure when extended fixation techniques, for example, transosseous fixation or biodegradable pin fixation, are impossible due to tight anatomical conditions or operational circumstances like the hip joint.

The presented technique is a single-step procedure. In a clinical study, Fontana et al. showed that the arthroscopic autologous chondrocyte transplantation (ACT) is superior to arthroscopic debridement of hip chondral defects [26]. However, ACT requires a two-stage operation which represents a burden for the patient. Combining our technique with an improved cartilage implant with Mfx, MSCs are attracted to immigrate into the bioinspired PM-scaffold. Thereby, bioinspired PM-scaffold depicts a matrix for the condrogenic differentiation of the MSCs.

In an arthroscopic hip study, Mancini and Fontana compared the clinical outcome of the arthroscopic matrix-induced autologous chondrocyte implant (MACI) with the autologous matrix-induced chondrogenesis (AMIC) techniques which is matchable to our technique [27]. The authors showed that the AMIC technique as a single-stage procedure is a valid procedure for improvement in patients concerned by chondral defects.

## 5. Conclusions

This study focuses on the arthroscopic application of enhanced cartilage implants on femoral hip cartilage lesions. Although this essential aspect is difficult to objectify, the authors of this paper underline the improved implantation conditions by using the PGA-PM-scaffold and eagerly anticipate the experiences of other arthroscopic hip surgeons. PGA-PM-scaffolds demonstrate increased observed initial fixation strength in cadaver femoral head defects relative to PGA-scaffold, particularly when fibrin glue is used for fixation. Further investigations including biomechanical adhesion analysis and studies with a greater number of cases are necessary.

## Figures and Tables

**Table 1:** Modified criteria for the scaffold evaluation described by Bekkers et al. [23]. The assigned points are given in brackets.

Outline attachment∗	Area coverage	Scaffold integrity∗∗	Endpoint fixation∗∗∗
Unchanged (5)	Unchanged (5)	Unchanged (5)	Cannot be detached (5)

<25% (4)	Shape deformities without structural damage (4)	Shape deformities or minor fissures that are unrelated to fixation (4)	Detached with intensive pull (4)

25–50% (3)	Fissures or cracks without important substances loss (3)	Minor fissures or cracks close to the fixation site (3)	Detached with minor pull (3)

50–75% (2)	<25% of scaffold lost (2)	Fissures or cracks endangering the fixation of the scaffold (2)	Detached with slight touch (2)

75–100% (1)	25–50% of scaffold lost (1)	Fissures or cracks endangering the fixation with surrounding scaffold disorganization (1)	Partial detachment (1)

100% (0)	>50% of scaffold lost (0)	Fissures or cracks endangering the fixation with generalized scaffold disorganization (0)	Total detachment (0)

∗% of full circumference that has lost contact with the surrounding cartilage rim.

∗∗% of total cartilage defect that is covered by scaffold.

∗∗∗The endpoint fixation was tested by a palpation hock manipulation after the motion cycles were completed.

**Table 2:** Results of two scaffold fixation techniques on two human cadavers after 50 cycles of continuous motion.

Material	Fixation technique	Outline attachment 50 cycles average score (±standard deviation)	Area coverage 50 cycles average score (±standard deviation)	Scaffold integrity 50 cycles average score (±standard deviation)	Endpoint fixation 50 cycles average score (±standard deviation)
PGA-scaffold	Unfixed	1.0 (±0)	1.5 (±0.7)	1.0 (±0)	0.5 (±0.7)

PGA-PM-scaffold	Unfixed	2.0 (±0)∗	2.0 (±0)	1.0 (±0)	2.0 (±0)∗

PGA-scaffold	Fixed with fibrin glue	2.5 (±0.7)	2.5 (±0.7)	3.0 (±0.7)	2.0 (±0.0)

PGA-PM-scaffold	Fixed with fibrin glue	3.5 (±0.7)∗	3.5 (±0.7)∗	4.0 (±0.7)∗	3.0 (±0.0)∗

Average per scoring item for the scaffold fixation techniques after 50 cycles (∗*P* < 0.05).

**Table 3:** Summary of the clinical procedure of the PGA-PM-scaffolds.

Advantages	Disadvantages	Pros	Cons
Single-stage procedure Suitable for medium chondral defects (2–4 cm²) Low operative cost	Demanding arthroscopic technique Demanding production of the implant	Minimal invasiveness procedure Rapid rehabilitation	Unsuitable for larger chondral defects (>4 cm²)
